# Determining a Likely Mechanism of Missingness in Repeated Measures Sleep Data From Wearable Fitness Trackers: Longitudinal Analysis

**DOI:** 10.2196/81123

**Published:** 2026-03-31

**Authors:** Kate Mobley, Kevin B Gittner, Karen Nielsen, Lauren M Matheny, Gita Taasoobshirazi, Charles Natuhamya, Monica H Swahn

**Affiliations:** 1 School of Data Science and Analytics, College of Computing and Software Engineering, Kennesaw State University, 680 Arntson Drive Atrium J333, MD 9104, Marietta, GA, 30060, United States, 1-470-578-2865; 2 Department of Health Promotion and Physical Education, Wellstar College of Health and Human Services, Kennesaw State University, Kennesaw, GA, United States; 3 Department of Population Health Sciences, School of Public Health, Georgia State University, Atlanta, GA, United States; 4 Uganda Youth Development Link, Kampala, Uganda; 5 School of Public Health, Virginia Commonwealth University, Richmond, VA, United States

**Keywords:** global health, wearable electronic devices, sleep, missing data, informal settlements

## Abstract

**Background:**

Wearable fitness trackers have become a valuable tool in public health research due to their ability to collect large-scale, individual-level data at a low cost. Because of this, wearables have the potential to mitigate the urgent need for health research in low-resource settings. However, their use has been largely limited to high-income settings, and a major challenge remains: high rates of missing data. This problem may be exacerbated in low-resource environments where logistical and operational barriers further complicate data collection. Wearable sleep data collected in an urban informal settlement in Uganda during The Onward Project on Well-being and Adversity project revealed substantial challenges related to missingness in low-resource research settings.

**Objective:**

The study aimed to characterize the patterns and frequency of missing data and to demonstrate the use of analytical methods to determine the underlying missingness mechanism.

**Methods:**

For this study, 300 women in slum communities in Kampala were equipped with Garmin smartwatches that collected data over 5 days. The nature of missingness was assessed through four methods: pattern analysis, the Little test, a random forest classification model, and a logistic regression classification model. Pattern analysis is an established method of identifying missing data patterns, while the Little test is used specifically to identify missing completely at random data. Random forest and logistic regression models are more recent methods proposed to identify the mechanism of missingness; both were used to examine agreement between the 2 methods and to ensure a thorough examination of missingness patterns.

**Results:**

Approximately 30% of nighttime data were missing. Three patterns were identified that occurred in over 10% of participants’ data: no data missing, fifth night of data missing, and all nights of data missing. Pattern analysis and the Little test (*P*<.001) indicated that the data were not missing completely at random. Both the random forest (area under the curve=0.7) and logistic regression models suggested that the data were missing at random (MAR).

**Conclusions:**

Evidence of missingness in this dataset was consistent with MAR. Potential causes of missingness include device removal, battery failure, and technical malfunctions. These findings have important implications for both wearable device users and future research. Understanding the mechanisms behind missingness can inform strategies to improve data quality, particularly in low-resource settings, and allow researchers to manage missingness using appropriate strategies designed to handle MAR data without introducing bias into the results.

## Introduction

### Wearable Fitness Trackers in Health Research

Originally intended for personal health and fitness tracking, wearable fitness trackers have gained traction in research due to their ability to collect large-scale, real-world data from participants in natural settings [1]. According to Fitabase, a platform monitoring research applications of Fitbit devices, over 1300 studies incorporated Fitbit devices between 2012 and 2025 [2] and that number increases when factoring in the studies that have used other fitness trackers. Wearable fitness trackers provide real-time data collection, including physical activity, heart rate metrics, biometric and health indicators, location, behavior, and sleep. These data are critical for identifying risk factors for various health conditions, tracking health outcomes over time, evaluating interventions, and advancing personalized medicine. Wearable device data, when limitations are managed, have the potential to advance understanding of how lifestyle behaviors interact with physical and mental health across diverse populations, supporting more effective, data-driven public health strategies and clinical practices. Despite their growing adoption in research, these technologies remain disproportionately used in high-income countries and among wealthier populations [1,3,4].

### Research in Urban Slum Communities

Wearable devices hold significant promise for research, particularly in low-resource settings such as urban slums [4,5]. These settings, defined by constraints across finances, health care, infrastructure, knowledge, research capacity, social resources, environmental factors, human resources, and cultural practices [6], pose unique challenges for data collection. Furthermore, over 1 billion people, or 24% of the world’s urban population, currently live in slums, with this figure rising to 56% in sub-Saharan Africa [7]. Wearables offer a practical solution to the need for health research in these settings with large populations and disproportionately high risks of physical and mental health conditions [8-10]. They are cost-effective, scalable, minimally intrusive, and capable of gathering individual-level data for multiple days without reliance on external power or additional technology. These advantages make them especially valuable for research in resource-limited environments [4,5]. Understanding missingness in sleep data from wearable fitness trackers can provide important foundational information for this research.

Understanding the physical and mental health challenges in urban slums and other low-resource settings is a critical public health priority, and sleep serves as a key indicator of overall well-being. Sleep problems, including poor sleep quality and short sleep duration, are common among residents of slum communities and have been linked to mental illness, but this topic is still understudied, especially so in low- and middle-income countries [4]. Wearable technology presents a transformative opportunity for such studies, offering cost-effective, minimally intrusive methods to collect longitudinal health data in challenging urban environments. For instance, while poor sleep is a documented issue in slums, existing research has relied primarily on subjective and unreliable self-reports rather than objective sensor-based measurements [4].

However, while missing data is an issue present in all research involving wearable fitness trackers, the difficulties associated with doing research in low-resource settings like urban slums can contribute to higher rates of missing data [11]. Residents of urban slums often have unreliable electricity access, are unfamiliar with wearable fitness tracker technologies, and face logistical barriers to implementing these technologies [12,13]. Additionally, many wearable fitness trackers are reliant on connection to a smartphone or the internet, and rates of internet access and smartphone usage are low in urban slums [4,14]. Because of these challenges associated with low-resource settings like urban slums, rates of missing data can be higher when wearable fitness trackers are used in these settings. Though wearable studies in slums remain limited and are often clinic-based, there is potential for scalable, real-world data collection using wearable devices [15-17]. To realize this potential, understanding critical challenges like missing data must become a priority.

### Missing Data

Missing data remains a pervasive challenge in human research, arising from diverse sources. Participant-related factors, such as missed study visits or attrition, and researcher limitations in follow-up capacity are well-documented contributors [18,19]. The growing integration of digital devices and sensors in research has introduced additional technical sources of missingness, including equipment malfunctions, battery failures, and connectivity issues [20]. Longitudinal studies, which require sustained data collection across multiple timepoints, face compounded risks of missing data, making this issue particularly acute in such designs [21].

Missing data in wearable device studies typically stems from two primary sources: device-related factors and wearer-related behaviors [22,23]. Technical issues such as battery failure, sensor displacement, software malfunctions, connectivity problems, or synchronization errors frequently contribute to device-related missingness [22-24]. Wearer-related missingness most commonly results from nonadherence, including device removal or improper wear [23]. There has been some study of missing data in wearable data, but very little in low-resource settings where wearables have only been used in a small number of studies outside of clinical settings, where conditions are much more controlled. Researchers in low-resource settings have theorized that a higher prevalence of manual labor in these settings results in exposure to extreme conditions, contributing to both device- and wearer-related challenges, which may contribute to the deterioration of wearable devices and increased missingness [5]. Wearables also differ widely in the types of data they collect, and only a few studies have considered missingness in wearable sleep data specifically [23,25]. Despite the growing use of wearable devices to collect sleep data, limited research has examined patterns and mechanisms of missingness, particularly in low-resource settings, where contextual challenges may further exacerbate missing data [11].

The prevalence of missing data significantly impacts data quality and poses substantial analytical challenges [26]. Effective handling of missing data through methods like imputation becomes crucial for ensuring accurate analyses [24]. A comprehensive review of wearable studies in biomedical research confirms that data missingness remains among the most significant obstacles researchers face [26,27]. Few researchers have used wearables in low-resource settings, but among those who have, numerous studies have found data missingness to be a substantial issue, underscoring the need to understand the mechanisms of missingness and to find robust solutions to this persistent issue [5,28].

Missing data can be categorized into three primary mechanisms: missing completely at random (MCAR), missing at random (MAR), and missing not at random (MNAR). When data are MCAR, the probability of missingness is entirely independent of both observed and unobserved data [29,30]. In contrast, MAR occurs when missingness depends only on observed variables, not the missing values themselves [30]. The most challenging scenario, MNAR, arises when missingness is related to the unobserved data [18,31]. Understanding these common classifications of missing data is necessary, as these distinctions depend on whether missingness is related to observed or unobserved information and has important implications for analysis and interpretation [29-31].

### Analytical Methods for Determining the Mechanism of Missingness

Determining the exact mechanism of missingness is challenging, but several methods can provide evidence for specific patterns. Visualization techniques offer a preliminary approach to identifying missingness patterns through graphical exploration [32]. For formal testing, the Little test, a well-established method, assesses whether data are MCAR [33]. To explore whether data are MAR, two analytical approaches are commonly used: random forest and logistic regression. Both methods model relationships between observed variables and a binary indicator of missingness. If significant associations exist between observed variables and missingness, this supports the MAR mechanism [34,35]. However, in longitudinal studies, where observations within participants are correlated, these methods require modifications to account for dependencies. In addition to contributing to the body of knowledge on missingness in wearable sleep data, this study demonstrates the use of these analytical methods on wearable sleep data, and on repeated measures data, where adjustments are necessary to preserve the dependencies between observations from the same person.

### Wearable Fitness Trackers in Sleep Research

Since around 2017, consumer wearable fitness trackers have measured time spent in each sleep stage using a combination of motion detection, heart rate variability, and respiratory rate monitoring [36]. While questions remain about the absolute accuracy of sleep-stage classification, the devices’ ability to detect sleep-wake patterns has significantly improved [37,38]. Importantly, wearable fitness trackers provide more objective sleep data than self-reports, as individuals often cannot accurately recall specific sleep metrics like sleep onset time or nighttime awakenings [36]. Although wearable sleep tracking technology still has limitations, it represents a substantial improvement over subjective reporting for research purposes, with accuracy continuing to advance through technological innovations [37-39].

Garmin devices like the ones used in this study have been assessed for the validity of their measurements. Existing studies indicate that they provide highly valid step count measurements, though their accuracy for distance, energy expenditure, and heart rate data remains more variable [40]. Garmin devices demonstrate reasonable agreement with polysomnography for measuring sleep duration and staging [41], and they more accurately identify deep sleep stages than some other wearable fitness trackers [42]. Manufacturers release new models frequently, and different models from the same brand can vary. Therefore, the comprehensive evaluation of wearable devices across all models and brands, including validity, reliability, proprietary algorithms, cost differences, and feature variability, remains challenging. Overall, however, modern wearable technologies that combine heart rate and motion tracking demonstrate comparable effectiveness for sleep monitoring, despite some variation in specific metrics across models and brands.

### Wearable Sleep Data Missingness

While the problem of missingness in wearables data, including sleep data, has been well documented, only 2 studies were identified that considered how to address this problem in sleep data [23,25]. However, these studies differed substantially from this work. First, both studies used sleep and wakefulness data collected every few seconds for up to 50 days, so that missingness was determined by measuring the length of time during the data collection period that no data was collected (a method typically referred to as measuring data completeness). Additionally, one of these studies used data from individuals with very irregular sleep patterns so that the results may be used by those studying sleep disorders, while the other removed participants who had removed the device at any point, a common cause of missing data [23,25]. Neither study used data from low-resource settings where missingness is likely to be even higher than in data from high-income populations.

### Study Purpose

The limited research on missing data in wearable sleep data that has been conducted so far uses sleep data that is in a significantly different format from that used in this study. To the authors’ knowledge, this is the first study to examine missingness in wearable sleep data aggregated by night within a low-resource setting. This innovative approach to analyzing missingness in wearable sleep data is closely aligned with the typical use and analysis of this type of data.

Wearable fitness trackers offer a promising tool for collecting sleep data in diverse environments, which can allow for further study of sleep and the impact of poor sleep on health. However, missing data remain a significant limitation, and the unique logistical challenges of conducting research in low-resource settings, where sleep and health research is urgently needed, may exacerbate this issue. Addressing the patterns of missingness is essential not only for improving data quality but also for advancing research on sleep’s role in health in all settings. This study has two primary objectives: (1) to characterize the patterns and frequency of missing data and (2) to determine the underlying missingness mechanism using analytical techniques appropriate for longitudinal repeated measures data. Enders [43] comprehensive work on missing data argues that MAR is the most reasonable assumption for the mechanism of missing data in most cases of research, and this assumption should be adopted unless there is evidence to the contrary. Therefore, this study will focus on evidence that the missingness is MCAR or MAR.

## Methods

### The TOPOWA Project

The data for this study were collected through The Onward Project on Wellbeing and Adversity (TOPOWA), a mixed methods investigation examining the social determinants of mental health among women in Kampala, Uganda’s urban slums. Details of the study have been reported previously [4,44]. Participant recruitment was conducted across 3 slum communities (Banda, Bwaise, and Makindye) in collaboration with the local community leaders and Uganda Youth Development Link, a nongovernmental organization serving youth in Kampala’s informal settlements.

TOPOWA project used multiple research components, including focus group discussions, Photovoice methodology, and a longitudinal cohort study which includes the collection of sleep data via wearable fitness trackers to study the prevalence of sleep disturbance and its association with mental health in this population.

### Ethical Considerations

All study procedures were designed in compliance with the Declaration of Helsinki and received ethical approval from both the Makerere University School of Social Sciences Research Ethics Committee (MAKSSREC MUSSS-2022‐172) and the Uganda National Council of Science and Technology (SS1560ES).

Uganda Youth Development Link staff at each study site described the TOPOWA study including risks, benefits, and expectations regarding follow-up assessments to interested participants. Informed consent in writing was obtained from those who agreed to participate in the study. Participants were compensated in the local currency for each component of the study that they participated in, including wearing and returning the wearable device and completing the survey, as well as being provided with food and transportation on the days that they traveled to the research hub to complete assessments. All research assistants involved in participant enrollment and data collection were trained in participant privacy, confidentiality, and safety measures. Data were deidentified before being uploaded to REDCap (Research Electronic Data Capture) for storage and shared with members of the research team for analysis.

### Data Collection and Processing

The TOPOWA project enrolled 300 women from Kampala’s urban slums, equipping each participant with a Garmin vívoactive 3 device to wear continuously for 5 days and nights. These devices were specifically selected for their field suitability: they operate independently without smartphone connectivity, feature a 5-day battery life, and automatically collect sleep data each night. During data collection, 299 devices were successfully returned, with only 1 unit lost. The vívoactive 3 devices record heart rate, heart rate variability, and movement data, which Garmin’s proprietary algorithms analyze to classify sleep into 4 stages (rapid eye movement [REM], deep, light, and awake) plus an “unmeasurable” category for periods that cannot be classified [45]. For each sleep session, the devices generate detailed outputs including the duration (in seconds) spent in each sleep stage and precise timestamps marking the beginning and end of the primary sleep period. Notably, when multiple sleep periods occur within a day, the system prioritizes and reports only the longest continuous sleep bout, overriding shorter naps. This design ensures researchers obtain standardized, comparable metrics for a person’s main sleep period, which will be discussed as nightly sleep for simplicity, while maintaining the practicality required for field studies in low-resource settings.

During the compilation of baseline sleep data, specific criteria were established for identifying missing data. These criteria focused on total sleep duration, treating nights with no sleep information as missing while retaining nights with vague or low-quality data. Notably, the absence of data for a particular sleep stage on nights when other stages were recorded was not classified as missing data. This pattern could indicate either that the participant genuinely did not enter that sleep stage or that the device failed to detect it—a distinction that cannot be determined from the available data. Consequently, 0 values for specific stages on otherwise recorded nights were treated as valid observations rather than missing data. No nights with data had a 0 recorded for light sleep, but there were nights that contained 0 for the deep sleep, REM sleep, and awake categories. Less than 2.5% of nights with data had a 0 recorded for multiple sleep stages. Similarly, “unmeasurable” sleep seconds were considered (periods the device could not classify into any stage) as legitimate data points and included in total sleep duration calculations, rather than being treated as missing values. Zeros were common in the “unmeasurable” category, indicating that there were no seconds of sleep that the device was unable to classify into one of the other sleep stage categories during those nights. This approach ensured that the analysis reflected the complete sleep architecture as captured by the device while maintaining methodological consistency.

The presence or absence of heart rate and activity data was also recorded. If heart rate data were present for the prior day, then a 1 was recorded, and if heart rate data were not present for the day before the night in question, then a 0 was recorded. If at least 1 activity was recorded during the 5 days that the participant wore the device, then a 1 was recorded for activity data, and if no activity was recorded over the 5 days, then a 0 was recorded. The presence or absence of heart rate data was used in the statistical analysis to determine the likely mechanism of missingness; however, since activity data was only collected during prolonged periods of physical activity and was therefore much less frequent, they were not included in the statistical models. While the Garmin sleep stage classification algorithm uses concurrently collected heart rate data, the missing data definition used in this study allowed for inclusion of nights with unclassified stages—therefore, the presence of heart rate data was not perfectly correlated with missingness.

### Statistical Analysis

The analysis of missing data followed a systematic approach. First, a pattern analysis was conducted using the *naniar* (version 1.1.0; Nicholas Tierney and Dianne Cook) package in R to visually examine the structure of missingness in the dataset [46]. Next, the Little test, a likelihood ratio-based test that compares the expected means for each variable across different missingness patterns [33], was applied to evaluate whether the data were MCAR. For the Little test, the data were converted to wide format, and the 5 sleep category variables (deep, light, REM, awake, and unmeasurable) were included as well as the variables for marital status, household size, education level, and employment status. This test was conducted first because the MCAR criteria are more restrictive than the MAR criteria.

Following this initial assessment, 2 distinct classification methods, a random forest model and a generalized estimating equations (GEE) model with a logit link function, were used to explore whether the missingness pattern was consistent with MAR. For both models, a binary dependent variable indicating whether data were present or missing for each night was created. Both models incorporated six predictor variables: (1) night sequence number (1-5), (2) household size, (3) education level, (4) employment status (binary), (5) marital status (binary), and (6) presence of heart rate data for the day before the night of sleep (binary). These covariates were selected based on established relationships between socioeconomic factors and sleep behaviors in prior research [47,48]. The results from both models were then used to strengthen the conclusions about the nature of the missing data mechanism.

The dataset included participants with complete missingness across all 5 nights of sleep monitoring, represented by NA (not available) values for all sleep stages. While standard practice might suggest listwise deletion for such cases, these participants were retained in this analysis for three key reasons: (1) they provided complete data for all independent variables (household size, marital status, education level, employment status, and heart rate data availability), (2) this study specifically aimed to develop alternatives to conventional listwise deletion approaches, and (3) missingness, even complete missingness, can be informative when evaluating the mechanism and patterns behind the missingness.

While the random forest classification and GEE modeling with logit link functions serve similar analytical purposes for MAR assessment, their concurrent application provides methodological robustness, given that (1) each approach has distinct strengths in handling different data characteristics and (2) their use for MAR determination remains relatively novel in the literature, with foundational publications emerging only in the early 2000s [34,35,49]. This dual-method approach strengthened the conclusions by assessing consistency across complementary analytical frameworks.

### Random Forest Classification Model

A random forest classification model was used using the *randomForest* R package (version 4.7.1.1; Andy Liaw and Matthew Wiener) to assess whether the data were consistent with MAR, as this approach can effectively capture complex, nonlinear relationships among observed variables [35]. Model performance was evaluated using the area under the receiver operating characteristic curve. An area under the curve (AUC) value significantly greater than 0.5 provided evidence for MAR, as this indicates that the missingness pattern was systematically related to the observed variables in the dataset [35]. Low AUC values (less than 0.5) indicate that the model performs no better than random guessing and would be expected if the data were MCAR. AUC values above 0.5 can occur if the data are MNAR but are more likely to occur if the data are MAR [35].

A critical consideration in the analysis was that standard random forest classification assumes independence between observations, an assumption violated in longitudinal data where multiple measurements come from the same individual. To address this dependency, a cluster-adjusted bootstrap sampling approach [50] was implemented, which resampled participants (rather than individual nights) to preserve within-subject correlations. While the use of a random forest model to assess evidence of MAR is not novel, modifying that model to account for dependencies between observations in repeated measures data is not a standard use of the method. For this reason, a second method of modeling patterns between observed variables was used.

### GEE Model With Logit Link Function

The second approach implemented for exploring a MAR mechanism was a logistic regression model. This method tests whether observed variables can systematically predict missingness patterns—a key criterion for MAR. While several logistic regression-based approaches exist for MAR assessment (including Ridout’s method for dropout analysis in longitudinal studies [34] and Fairclough’s method for missingness prediction), Fairclough’s framework [34] was adapted for this analysis since its purpose was closely aligned with the goal of this study. Independent variables were entered sequentially into the model to assess their association with missingness. A variable’s contribution was evaluated based on the regression coefficient estimates, with statistical significance determined using robust standard errors derived from the GEE, to characterize predictors of missingness. Five total models were created with an additional variable added in each model. This iterative approach using forward selection allowed for assessing how different participant characteristics and study factors influenced the probability of data being missing for a given night.

While traditional logistic regression assumes independence between observations, this assumption is violated in repeated measures designs where observations from the same participant are inherently correlated [51]. To address this dependency, a GEE model with a logit link function was used using the *geepack* package in R (version 1.3.12; Søren Højsgaard, Ulrich Halekoh, Jun Yan, and Claus Thorn Ekstrøm), which extends generalized linear models by incorporating specified covariance structures [52]. Working correlation structures were assessed for fit, including the unstructured, exchangeable, and autoregressive (1). The quasi-likelihood under the independence model information criterion was used to assess which working correlation structure was most suitable [52]. For this analysis, an exchangeable working correlation structure was selected, which assumes that the correlations between observations from the same participant are equally correlated [52]. This selection is appropriate because the data were collected over just 5 consecutive days, so significant correlation decay is not expected in such a short time period. The GEE framework allowed for properly accounting for within-participant correlations while examining how various factors predicted missingness patterns.

While the GEE model with a logit link function and the random forest model perform similarly in that they both model relationships between observed variables, neither is a standard method of assessing the mechanism of missingness that has been well documented in previous publications, as is the case for the Little test. Literature was identified that supported the validity of these methods, but no prior studies using these methods were identified. For these reasons, both modeling methods were performed so that their results could be compared.

## Results

### Participant Characteristics

All women who participated in the TOPOWA project were aged between 18 and 24 years old. All participants had completed some primary school and could read and write, but only 8% (24/300) had completed secondary school. Over half, 62% (186/300), had given birth and 82% (152/186) of those who had given birth lived with her child(ren). Only 15% (45/300) of the women lived in a single generation household, while the remaining 85% (255/300) lived in households with 2 or more generations. Women who had a job represented 32% (97/300) while the remaining 68% (203/300) of the women were unemployed.

### Missing Data in the TOPOWA Baseline Sleep Data

Participants wore Garmin devices continuously for 5 days and nights, yielding sleep data from 299 individuals. However, 37 (12%) devices recorded no sleep data across all 5 nights (complete missingness). The dataset exhibited a notable missing data pattern: nights were either fully observed (all sleep stages recorded) or fully missing (no data for any sleep stage). Consequently, missingness proportions were identical across all sleep metrics (deep, light, REM, awake, and unmeasurable seconds), with an overall missing data rate of 30% (455 nights missing out of 1495 total nights). The rate of missingness was similar across the 3 study sites (Banda, Bwaise, and Makindye) with Banda having a slightly higher rate of missingness than Bwaise and Makindye (Table 1). The rate of missingness in the sleep data remained consistent as household size increased but increased slightly with each additional child a woman had, so that participants with no children had the lowest rate of missingness (155/570 nights of data, 27%) and participants with 3 children had the highest rate of missingness (18/25 nights of data, 72%) (Table 1). The rate of missingness in the sleep data was approximately equal across changes in income, employment status, education level, and marital status. Notably, 58% (173/299) of participants experienced at least 1 night of missing data, demonstrating the pervasiveness of this issue.

**Table 1.** Proportion of missing data by study site, night of data collection, and number of children.

Rate and number of nights of data missing	Nights of data (n=495) missing from Banda participants, n/N (%)	Nights of data (n=500) missing from Bwaise participants, n/N (%)	Nights of data (n=500) missing from Makindye participants, n/N (%)	Total nights of data (n=1495) missing from all participants, n/N (%)
Night of data collection				
1	22/99 (22)	25/100 (25)	20/100 (20)	67/299 (22)
2	32/99 (32)	26/100 (26)	21/100 (21)	79/299 (26)
3	32/99 (32)	25/100 (25)	24/100 (24)	81/299 (27)
4	35/99 (35)	21/100 (21)	32/100 (32)	88/299 (29)
5	49/99 (49)	44/100 (44)	47/100 (47)	140/299 (47)
Number of children				
0	34/155 (22)	65/155 (42)	56/155 (36)	155/570 (27)
1	89/188 (47)	45/188 (24)	54/188 (29)	188/625 (30)
2	35/94 (37)	26/94 (28)	33/94 (35)	94/275 (34)
3	11/18 (61)	7/18 (39)	N/A^a^	18/25 (72)

a N/A: not available.

The 37 cases of complete missingness for all 5 nights of sleep data can be categorized into 3 distinct scenarios with varying probable causes, detailed in Table 2.

**Table 2.** Categorization of the 37 cases of complete sleep data missingness.

Status of sleep and heart rate data	Number of cases
No sleep or heart rate data collected	1 device
Partial data collection—either heart rate or activity (but not both) data collected for at least 1 day	8 devices
Selective sleep data missingness—devices collected both heart rate and activity data for at least 1 day, but no sleep data	28 devices

### Data Exploration

The distributions of time spent in each sleep stage and total time asleep were examined to determine if the distributions differed based on the day of the data collection (1-5). For each sleep stage, as well as total time asleep, the distribution was the same both when the 5 nights of data were considered together and when the data were grouped by night. There were no changes in the distribution of sleep times over the course of the 5 days of data collection.

There were some outliers in the total sleep durations. For 1 participant, less than 1 hour of sleep was recorded in 1 night, and for another participant, just over 12.5 hours of sleep was recorded on 1 night. Because the outlier values present in the data are not outside the realm of possibility, these observations were retained when assessing the missing data mechanism.

### Missingness Patterns

**Figure 1.**
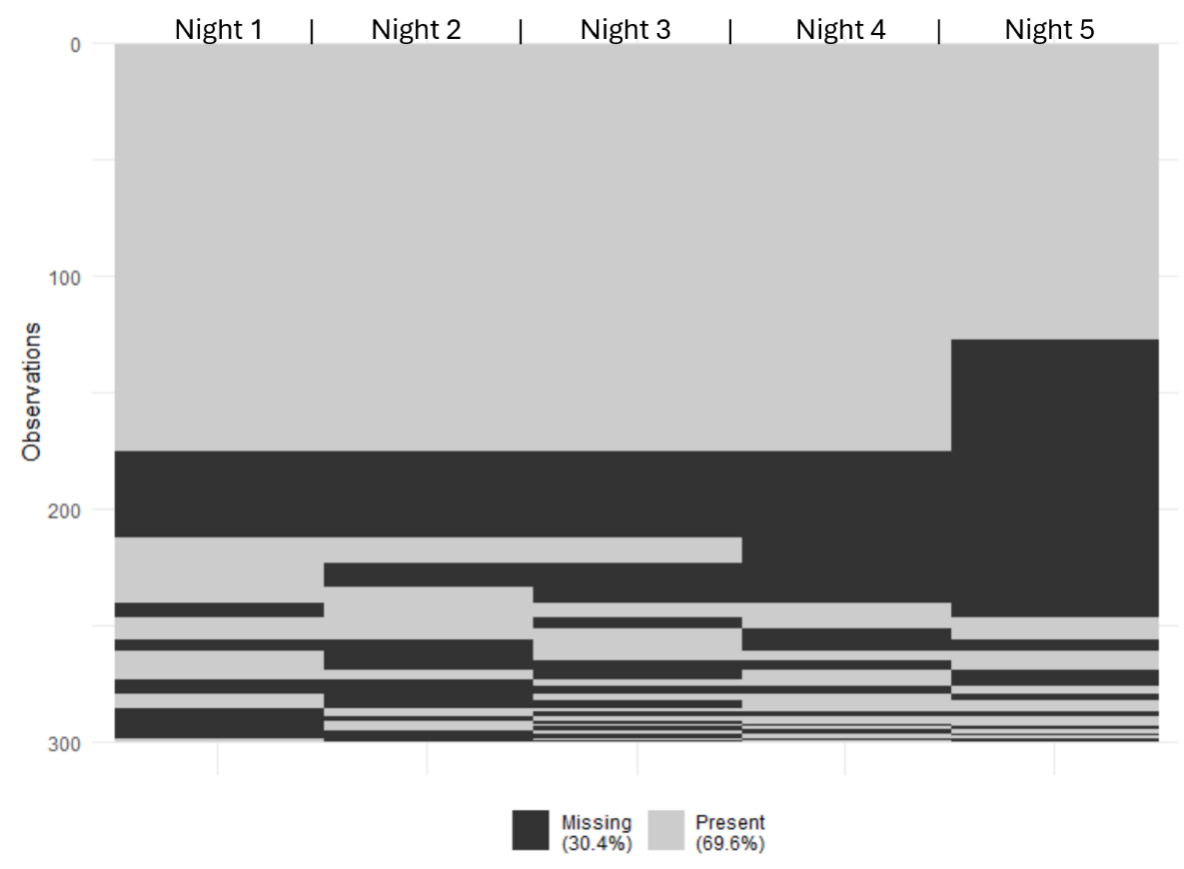
Patterns of missingness in the sleep data. Missingness patterns are ordered from most common at the top (no missing nights of data) to the least common (bottom).

The first night of data collection has the least amount of missing data, with 22% (66/300) of nights missing data. The rate of missing data increases for each subsequent night, and the fifth and final night of data collection has 47% (141/300) of data missing (Table 1). There were 29 missingness patterns identified in the data (Figure 1), with no nights missing being the most common (126 cases), followed by missing only the last night of data (48 cases) and then missing all nights of data (37 cases).

### The Little Test

When running the Little test, the null hypothesis is that the data are MCAR while the alternative hypothesis is that the data are not MCAR and are therefore either MAR or MNAR. The *χ*^2^ statistic was 969 and the degrees of freedom was 587, which resulted in a *P* value for the Little test of *P*<.001. Therefore, the null hypothesis is rejected. This suggests that the data were not MCAR.

### Random Forest Classification Model

The AUC value of 0.70 for the random forest classification model indicated that the model was generally able to distinguish missingness, which is consistent with data MAR. The sensitivity, a measure of how well true positives (ie, missing data) are predicted, was 0.30 and the specificity, a measure of how well true negatives (ie, data present) are identified, was 0.94. The overall accuracy for the model was 0.75. Table 3 shows the confusion matrix for the random forest classification model.

**Table 3.** Random forest classification model confusion matrix.

Predicted values	True values	
	0 (data present)	1 (data missing)
0 (data present)	978	319
1 (data missing)	62	136

### GEE Model With Logit Link Function

The results of the GEE model with a logit link function also yielded results that were consistent with a MAR assumption. The independent variables were added to the model in an iterative process to determine the extent to which each of the variables was able to predict missingness. The final model (Table 4) contained only 2 predictor variables that were statistically significant, number of night in the 5-night sequence (1-5) and whether the participant had a job. The model had an accuracy of 0.70. The sensitivity was 0.12, while the specificity was 0.96. The confusion matrix for the model is displayed in Table 5.

**Table 4.** Results of generalized estimating equations model with logit link function.

	Parameter estimate	SE	Odds ratio (95% CI)		*P* value
Intercept	–1.8546	0.1850	0.156 (0.109‐0.225)		<.001
Number of night in the 5-night sequence	0.2554	0.0357	1.291 (1.204‐1.384)		<.001
Job	0.6671	0.2016	1.948 (1.312‐2.893)		.001

**Table 5.** Confusion matrix for the generalized estimating equations model with logit link function.

Predicted values	True values	
	0 (data present)	1 (data missing)
0 (data present)	996	402
1 (data missing)	44	53

### Comparing the Results of the 2 Classification Models

The random forest model and the logistic regression model agreed in 90% (1346) of cases. Table 6 contains the confusion matrix showing how often the 2 models agreed and how often they disagreed in their classifications.

**Table 6.** Confusion matrix comparing the random forest and logistic regression models.

Logistic regression predictions	Random forest predictions	
	0 (data present)	1 (data missing)
0 (data present)	1281	117
1 (data missing)	32	65

## Discussion

### Principal Results

Our comprehensive analysis provides evidence that the missing sleep data in the TOPOWA study follows a MAR pattern, thereby permitting the use of imputation and likelihood-based methods that would be inappropriate under an MNAR mechanism. Furthermore, Enders [43] recommends adopting MAR as the default assumption in most realistic research scenarios. The findings in this study indicate that missingness was systematically related to observable variables rather than unobserved sleep characteristics [21,53]. Both the random forest model and the GEE model with a logit link function had high specificity values, indicating that the models identified true negatives well (present data values), but the values for sensitivity were low, indicating that the models performed worse at identifying true positives (missing data values). The classification models more accurately identified when data values were present than when data values were missing. However, previous researchers who have used this method to check for evidence that missing data are MAR have focused on the AUC value and did not report sensitivity and specificity [35].

While additional studies using varying types of data, such as supplementary self-reported sleep data, are necessary to further validate the methodology used in this study, the results suggest that a GEE model with a logit link function and a random forest classification model can be used to identify evidence that missing data are MAR in repeated measures data when modifications are made to account for the dependencies between observations from the same person. This finding has the potential to improve researchers’ handling of missing data in longitudinal data, common in health research, by providing a way to empirically determine the likely mechanism of missingness prior to analysis and decide how best to handle the missing data based on that mechanism of missingness.

### Possible Explanations for Missing Data

Several key observations support the conclusion that the missingness is MAR. First, the temporal pattern of missingness showed a clear progression, with later nights (particularly night 5) having higher rates of missingness than earlier nights. Second, known explanations for missingness—including device failure (n=1 likely case), nonadherence (n=3 self-reported cases), or improper device fit—would generally qualify as MAR mechanisms. While 37 participants had no sleep data for any of the 5 nights, the available evidence suggests these cases resulted from technical or behavioral factors rather than being related to the missing sleep values themselves.

Missing data in the baseline sleep dataset likely stemmed from multiple sources, each with distinct implications for analysis. Battery depletion emerged as a primary factor, with some devices failing to maintain charge through all 5 nights, according to reports from the research assistants. This explanation aligns with the observed increase in missingness across successive nights and qualifies as MAR, as battery failure was unrelated to participants’ sleep patterns. The most common missingness pattern in the data was data missing on the fifth night only, which likely occurred because the battery was depleted before the fifth night. The third most common missingness pattern was data present for the first 3 nights and missing for the fourth and fifth nights, which also likely occurred due to a depleted battery. A subset of cases involved unpredictable device malfunctions (MCAR), where hardware failures caused complete data loss independent of user behavior or sleep characteristics. This was likely the case for the 1 participant for which no heart rate, activity, or sleep data was collected, and may have been the case for the 8 participants for whom either heart rate data or activity data was collected (but not both) and no sleep data was collected. Another possibility is that the missing data in the underlying raw data (heart rate variability and motion data) resulted in insufficient physiological data for sleep algorithm processing. This pattern highlights the technical and behavioral challenges inherent in wearable research, where algorithm-derived outcomes (like sleep duration and stages) depend on sensor data.

Participant-related issues also contributed: some women reported removing devices due to social discomfort (eg, partner or child concerns) [14], while improper fit (eg, loose wrist placement) prevented sensor contact, mimicking nonwear. In the case of the 8 participants for whom no sleep data was collected but either heart rate or activity data was collected, and the 28 participants for which heart rate and activity data was collected but sleep data was not, intermittent device removal (ie, at night) may have been a factor contributing to sleep data missingness. The coexistence of these mechanisms, and the potential for both technical and behavioral reasons for missingness, highlights the complexity of missingness in wearable research and underscores the need for robust analytical approaches to mitigate bias.

While only 3 women reported removing the device for any period of time during the 5-day data collection period, the missingness patterns in the data from considerably more devices suggested that device removal was a possible factor. The second most common missingness pattern in the data was data missing for all 5 nights, which could indicate the participants removed the device. Notably, the discrepancy between self-reported and probable nonwear suggests possible social desirability bias, as participants received compensation for participation.

### Limitations

Several important limitations should be considered when interpreting this study’s findings. The presence or absence of other physiological or behavioral metrics that wearable devices can capture (eg, activity levels, heart rate variability) was examined, but only the presence or absence of this data was tracked. Detailed information on heart rate or activity was not analyzed. Low-quality data may also mask data that should have been marked as missing. Some extreme sleep duration values (both small and large) were identified that could represent either genuine biological variation or measurement artifacts. Wearable devices may misclassify wakefulness as sleep during prolonged sedentary periods or conversely interpret restless sleep as wakefulness. While these devices generally provide more objective sleep measurements than self-reports, such classification errors underscore the importance of interpreting extreme values cautiously. Future studies could benefit from incorporating complementary data sources such as self-reported sleep to validate these outlier observations.

While it is possible that the day of the week may impact missingness, this was not explored in this analysis for 2 reasons. The women in this study were issued Garmin devices for 5-day periods on a rolling basis, meaning that not all women wore the devices over the weekend. The second reason that considering weekday versus weekend missingness is not ideal using this data is that the lifestyle and employment status of these women are very different from those of middle- and working-class populations, as well as Western assumptions of a structured 5-day work week, who typically have consistent and differing routines on weekdays and weekends. Most of these women did not have jobs with regular Monday-Friday hours.

Lastly, questions regarding the validity and reliability of consumer-grade wearable devices remain and should be further researched, especially because validity can vary between different device models from the same brand using similar technology. As health research using wearable devices continues and expands, the study of the validity of these devices should continue as well, especially as new models and technology that have not been previously studied are released. Additionally, self-reported sleep data has long been used for sleep research and can be a valuable tool when used as complementary data for validating sleep data from wearable technology.

### Conclusion

This work demonstrates the process researchers can undertake when assessing missing data assumptions prior to the analysis of wearable data. Taken together, these findings suggest that a MAR mechanism can be assumed for sleep data in the TOPOWA study, with missingness driven primarily by observable technical and behavioral factors rather than unobserved sleep characteristics. This distinction is critical for selecting appropriate imputation strategies and ensuring valid inferences in future analyses. As wearable methods expand in global health research, especially in urban informal settlements, addressing the practical and ethical complexities of data missingness will be essential for advancing equity, rigor, and reproducibility. In addition to considering data collected in a low-resource setting, where research faces additional challenges, this study contributes meaningful information on missingness in wearable sleep data aggregated by night, closely aligning it with the common use and analysis of this type of data.

## Abbreviations

AUC: area under the curve
GEE: generalized estimating equations
MAR: missing at random
MCAR: missing completely at random
MNAR: missing not at random
REDCap: Research Electronic Data Capture
REM: rapid eye movement
TOPOWA: The Onward Project on Well-being and Adversity
